# Ultra-Low Power CMOS Logic and Photodetection in WSe_2_ Semiconductors by Selective Area Plasma Doping

**DOI:** 10.1021/acsami.6c04479

**Published:** 2026-06-22

**Authors:** Jheng-Jie Lin, He-Yu Chen, Sheng-Zhu Ho, Xiaofan Lin, Greg Chu, Faris Abualnaja, Zhen-You Lin, Yu-Chiang Hsieh, Kuo-En Chang, Chung-Lin Wu, Kenji Watanabe, Takashi Taniguchi, Yi-Chun Chen, Tse-Ming Chen, Jack A. Alexander-Webber, Luke W. Smith

**Affiliations:** † Department of Physics, 34912 National Cheng Kung University, Tainan 701, Taiwan; ‡ Center for Quantum Frontiers of Research & Technology (QFort), National Cheng Kung University, Tainan 701, Taiwan; § Academy of Innovative Semiconductor and Sustainable Manufacturing, National Cheng Kung University, Tainan 701, Taiwan; ∥ Electrical Engineering Division, Department of Engineering, 2152 University of Cambridge, Cambridge CB3 0FA, U.K.; ⊥ Research Center for Electronic and Optical Materials, 52747 National Institute for Materials Science, 1-1 Namiki, Tsukuba 305-0044, Japan; # Research Center for Materials Nanoarchitectonics, National Institute for Materials Science, 1-1 Namiki, Tsukuba 305-0044, Japan; a Department of Materials, Loughborough University, Loughborough LE11 3TU, U.K.

**Keywords:** two-dimensional, transition metal dichalcogenides, tungsten diselenide, oxygen plasma, p-type doping, work function, local capacitance gradient

## Abstract

Among two-dimensional semiconductors, tungsten diselenide (WSe_2_) is particularly suitable for monolithic complementary metal-oxide-semiconductor (CMOS) architectures due to its tunable carrier polarity. Surface oxidation has emerged as a simple and effective route to control polarity and enhance p-type behavior; however, despite its widespread use, fundamental characterization of the oxide-induced electrostatic landscape in WSe_2_ remains limited. Here, we employ surface oxidation and selective-area oxide removal to form a lateral junction within few-layer WSe_2_. We apply a modified broadband electrostatic force microscopy technique to map nanoscale capacitance variations, providing contact-free insight into local carrier dynamics analogous to conventional MOS *C*–*V* measurements, probing oxide-induced electronic effects beyond conventional spectroscopy methods. Bias-dependent measurements reveal a distinct p–n junction between oxidized and oxide-removed regions, with a built-in surface potential difference of approximately 120 meV measured by Kelvin probe force microscopy, demonstrating spatially controlled polarity engineering without heterogeneous material integration. Field-effect transistors fabricated on the n- and p-regions integrated into a complementary inverter circuit exhibit ultralow peak static power consumption ∼13 pW at *V*_D_ = 1 V, among the lowest reported for WSe_2_-based logic circuits. We demonstrate self-powered photovoltaic photodetection resulting from the in-built electric field of the p–n junction, with fast, reproducible submillisecond response, symmetric rise and fall times, linear current–power dependence, and minimal charge trapping—key metrics for robust and reliable photodetection. Together, these results establish oxide-defined lateral p–n junctions in WSe_2_ as a compelling platform for reliable, ultralow-power electronic and optoelectronic applications.

## Graphical abstract

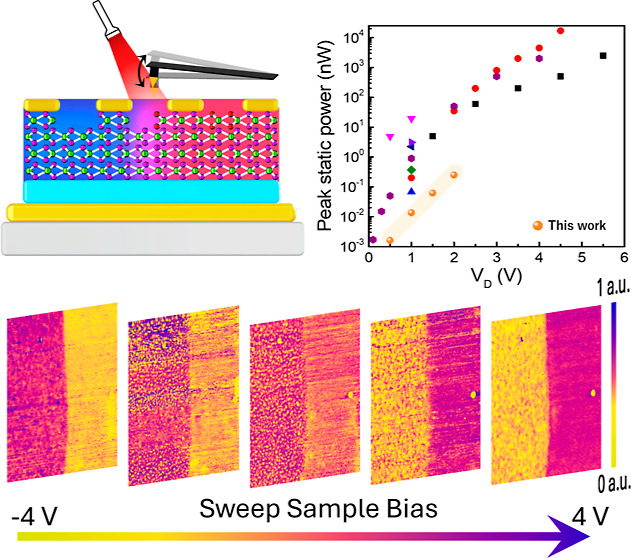

## Introduction

Two dimensional (2D) transition metal dichalcogenides (TMDs) have attracted significant attention for future transistors, driven by their potential for ultrathin body and subnanometer gate lengths,^1,2^ and where the absence of surface dangling bonds and short-channel effects provides significant advantages over traditional silicon for device miniaturization.^3–6^ The development of 2D complementary metal-oxide-semiconductor (CMOS) logic has become a major focus^7–9^ requiring practical integration of n- and p-type transistors in a single platform, and as computing demands continue to grow, ultralow power consumption and energy efficiency are central goals for 2D electronic systems.

Molybdenum disulfide (MoS_2_) and tungsten diselenide (WSe_2_) are typically employed as n- and p-type channel materials, respectively, and advances toward 2D CMOS architectures have been achieved through heterogeneous integration of distinct materials for the n- and p-channels.^10–13^ However, homogeneous CMOS based on a single 2D material^8^ offers significant advantages by simplifying material synthesis and streamlining fabrication, attractive for scalable 2D logic technologies.^14^ In this context, WSe_2_ is particularly attractive because its carrier polarity can be tuned through multiple approaches, including chemical surface treatments,^15^ controlled oxidation,^16–18^ contact engineering,^19–21^ the work function of source–drain metals,^22^ electrostatic gating,^23^ dielectric environment,^8^ and thickness modulation.^8,19^ Approaches that electrostatically control carrier polarity using split-gate geometries—including gate-tunable contacts,^12,24^ channel-defined logic elements,^25^ and reconfigurable p–n junctions^23,26,27^ —are attractive for reconfigurable device architectures, as they enable precise, in situ, and reversible control of carrier polarity. However, these methods typically require continuous gate biasing, increasing the static power consumption and complicating circuit-level integration and scalability due to the need for multiple independently addressable gate electrodes. In contrast, selective-area doping as presented here enables the formation of permanent lateral junctions without continuous gate biasing, thereby reducing power consumption and simplifying device architecture. This offers improved scalability due to reduced fabrication complexity and the absence of complex gate routing.

In particular, surface oxidation is attractive due to its process simplicity and effectiveness in strongly enhancing p-type behavior in WSe_2_.^17,18,28–30^ In this approach, a self-limited tungsten oxide (WO_*x*_) layer induces strong hole accumulation in the underlying WSe_2_ through surface charge-transfer doping (SCTD).^16,31^ However, despite its prevalence, previous studies almost exclusively focus on device performance, while fundamental characterization of the oxide-induced electrostatic landscape remains limited. We employ controlled oxidation and selective etching to form a lateral p–n junction within a single WSe_2_ flake and resolve a work function difference of ∼120 meV using Kelvin probe force microscopy (KPFM).^32^ We further apply a modified broadband electrostatic force microscopy (bb-EFM) technique^33^ to quantitatively map nanoscale capacitance variations at microwave frequencies, which has not previously been demonstrated for 2D material systems. Bias-dependent measurements provide insight analogous to conventional MOS *C*–*V* characterization of the local carrier dynamics, elucidating differences in the local majority carrier type and relative doping level between regions. This approach avoids the influence of metal contacts, where the measured polarity can be influenced by metal–semiconductor interface effects such as Fermi-level pinning.^19^

These measurements establish oxide-removed few layer WSe_2_ regions as n-type and oxide-covered regions as p-type, forming a lateral p–n junction. Integrating n- and p-FETs fabricated on each region into an inverter circuit shows a record-low peak static power consumption of ∼13 pW for WSe_2_-based inverters at *V*_D_ = 1 V. Furthermore, the built-in electric field of the p–n junction enables self-powered photodetection in the photovoltaic regime, exhibiting robust and fast submillisecond response with minimal charge trapping, demonstrating clear advantages for reliable, ultralow-power optoelectronic applications.

## Results and Discussion

### Surface Potential and Local Carrier Contrast

A simplified schematic of the device assembly for surface microscopy measurements is shown in Figure 1(a), further details are given in the Methods section. Graphite provides a conductive substrate required for biasing the sample. Four-layer WSe_2_ is used for all devices in this study, where layer thickness is confirmed by photoluminescence (PL) spectroscopy, Supporting Information Figure S1. A low-power (10 W) room-temperature oxygen plasma treatment of the entire flake induces controlled surface oxidation,^17,18,28,34^ leading to strong p-type conduction^19,35–38^ through SCTD.^16,31,39^ Photoluminescence and Raman measurements, Supporting Information Figures S1 and S2, are consistent with oxidation of the uppermost WSe_2_ layer.^16–18,28,29,31,35,40,41^ This full-surface oxidation approach protects the underlying WSe_2_ during subsequent lithographic processing.^42^ The oxide is then selectively removed using a potassium hydroxide (KOH) etch in areas defined by a poly­(methyl methacrylate) (PMMA) mask, leaving a clean, high-quality WSe_2_ surface^42,43^ without direct exposure to resist residues.

**Figure 1:**
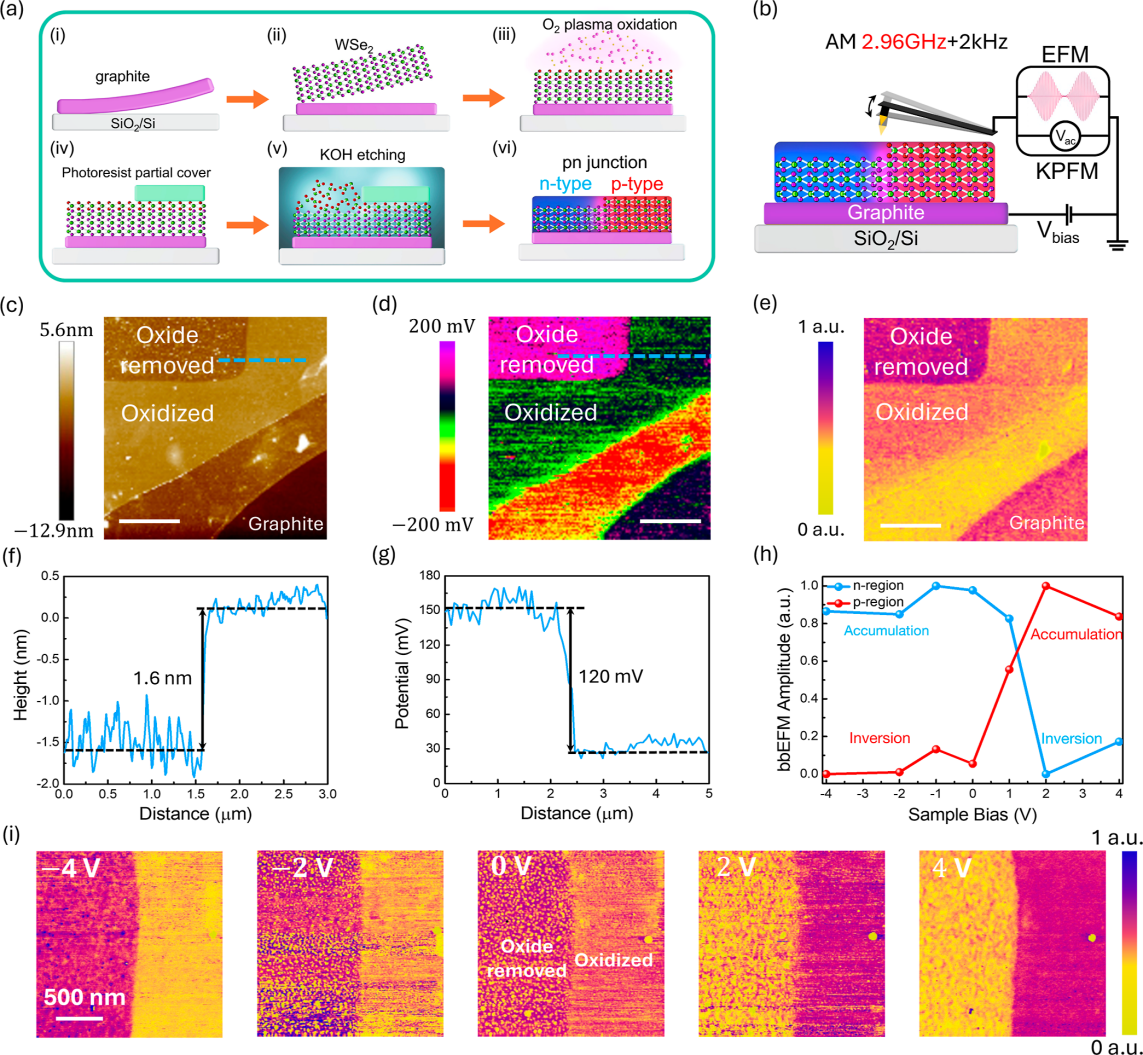
Surface potential and local capacitance properties of selectively oxidized WSe_2_. (a) Fabrication process of the WSe_2_ oxidized-pristine junction designed for surface measurements. (b) Schematic illustration of the KPFM and bb-EFM measurement set up. (c–e) AFM topography, surface potential measured by KPFM, and bb-EFM mapping, respectively. The entire WSe_2_ flake is oxidized and the oxide selectively removed in regions as marked. The scale bars in (c), (d), and (e) correspond to 2 μm. (f) Height profile corresponding to the blue dashed line in (c), showing a WO_*x*_ thickness ∼1.6 nm. (g) Potential profile corresponding to the blue dashed line in (d), showing a potential shift of ∼120 mV between oxidized and oxide-removed regions. (h) Bias-dependent bb-EFM spectra and (i) corresponding maps acquired across the WO_*x*_/WSe_2_ lateral junction in (e), indicating the local capacitance variation. The scale bar in (i) corresponds to 0.5 μm.

We quantify the lateral junction using KPFM^32^ and bb-EFM^33^ techniques, to directly measure the surface potential and local carrier contrast of the oxidized and oxide-removed regions. While oxidized WSe_2_ has been optically characterized using techniques such as X-ray photoelectron spectroscopy (XPS) and PL, quantitative understanding of the capacitance modifications and spatial electrostatic potential variations induced by surface oxidation has been lacking. A schematic diagram of the experimental set up is shown in Figure 1(b). We measure the surface potential and the surface morphology of the WSe_2_/graphite heterostructure, where the graphite serves as the bottom electrode. Figure 1(c) shows the AFM topography of the sample. The central region represents the oxidized region, as labeled. The region where oxide has been selectively etched is labeled as oxide removed and corresponds to three-layer WSe_2_. The bottom-right area corresponds to the exposed graphite bottom electrode, and the diagonal stripe is a thinner layer of oxidized WSe_2_ at the flake edge. Figure 1(f) shows the height profile corresponding to the blue dashed line in Figure 1(c), revealing a uniform WO_*x*_ thickness of approximately 1.6 nm on the unetched WSe_2_, consistent with previous reports.^16^ Figure 1(d) shows the corresponding surface potential measured by KPFM, displaying a pronounced contrast between oxidized and oxide-removed regions; therefore, a distinct work–function difference across the interface is observed. This local potential variation originates from the Fermi-level realignment resulting from the oxide layer creating the lateral p–n-like junction within a single flake. The potential profile corresponding to the blue dashed line in Figure 1(d) is shown in Figure 1(g), showing a work function difference of ∼120 meV.

Although KPFM provides direct information on the static surface potential, it cannot fully capture the dynamic capacitance variation or local carrier modulation under an external bias, as is necessary to determine differences in carrier type or density across the junction. To address this limitation, we implemented modified bb-EFM—an advanced tapping-mode measurement based on a sideband detection scheme—which enables simultaneous quantitative high-spatial-resolution mapping of the bias-dependent d*C*/d*z* at microwave frequencies.^44–47^ This technique employs an amplitude modulation excitation in which a radio frequency (GHz) excitation signal drives carrier oscillation, while a low-frequency (kHz) modulation detects the capacitance-force response.^33^ The bb-EFM technique is nondestructive by utilizing tapping-mode operation, and the frequency can be tuned to match carrier dynamics in 2D materials. This technique quantitatively distinguishes carrier polarity (n- versus p-type) and due to its sensitivity to carrier density is applicable to differentiating between regions such as n and n^+^ or p and p^+^. In contrast, conventional AFM-based carrier measurements are performed using scanning capacitance microscopy (SCM), where the contact-mode operation risks damaging 2D materials. Additionally, a metal-insulator-semiconductor structure is required and is unsuitable for oxide-removed samples studied here.

Figure 1(e) shows the nanoscale capacitance variation mapped by bb-EFM at an applied bias of −4 V, reflecting the locally tuned carrier distribution. Figure 1(i) shows the bb-EFM mapping of the junction behavior with sample bias from −4 to 4 V, which captures the evolution of d*C*/d*z* amplitude as a function of applied bias and reveals a pronounced carrier contrast across the WO_*x*_/WSe_2_ interface, resulting from variations in carrier density. Figure 1(h) shows the mean value of both oxidized (red trace) and unoxidized (blue trace) regions extracted from Figure 1(i) as a function of sample bias. Opposite trends are observed for both regions indicating opposite polarity, where, for the case of the WO_*x*_-covered region, the semiconductor transitions through inversion, depletion, and accumulation as the sample bias increases, indicative of a p-type semiconductor, and vice versa for the oxide-removed (n-) region. Note that the positive terminal of the sample bias is applied to the bottom electrode; therefore, the trends are opposite to the conventional MOS structure *C*–*V* curve.^48^ These results confirm the interpretation of the oxidized WSe_2_ region as a p-doped domain and demonstrate that oxygen-plasma treatment provides reliable and spatially selective control over charge polarity, while bb-EFM measurements provide insight into local carrier dynamics and interfacial capacitance properties of 2D semiconductors. We also note that the bb-EFM is contact-independent and thereby measures intrinsic material properties and provides insight that cannot be gained from electrical measurements alone, where contact effects such as Fermi-level pinning significantly affect the measured polarity.

### FET and Inverter Performance

Separate devices incorporating a local back-gate are fabricated to quantify the electrical performance of the junction. A simplified schematic of the device assembly process is shown in Figure 2(a), with further details given in the Methods section. The back gate electrode is created by a locally evaporated Cr/PdAu back gate overlaid with hBN, providing an atomically flat dielectric surface and mitigating the impact of surface defects and trapped charge at the SiO_2_ substrate.^49,50^ The oxidized and oxide-removed areas are designated as the p- and n-regions, respectively, as shown in Figure 2(b). Transfer characteristics of the pristine four-layer WSe_2_ are given in Figure 2(c), showing an ambipolar behavior. After the oxidation and oxide removal steps, transfer characteristics in Figure 2(d) show n-type behavior in areas with the oxide removed and p-type behavior in regions with the native oxide, confirming effective polarity control revealed by the bb-EFM measurements. The origin of the n-type behavior following KOH etching may potentially arise from contact effects and/or process-induced modifications of the electronic properties.^51^ In the oxidized region, improved hole injection at the contacts also contributes to strong p-type behavior, where contact-adjacent oxidized regions modify the height and width of the Schottky barrier.^18,28^

**Figure 2:**
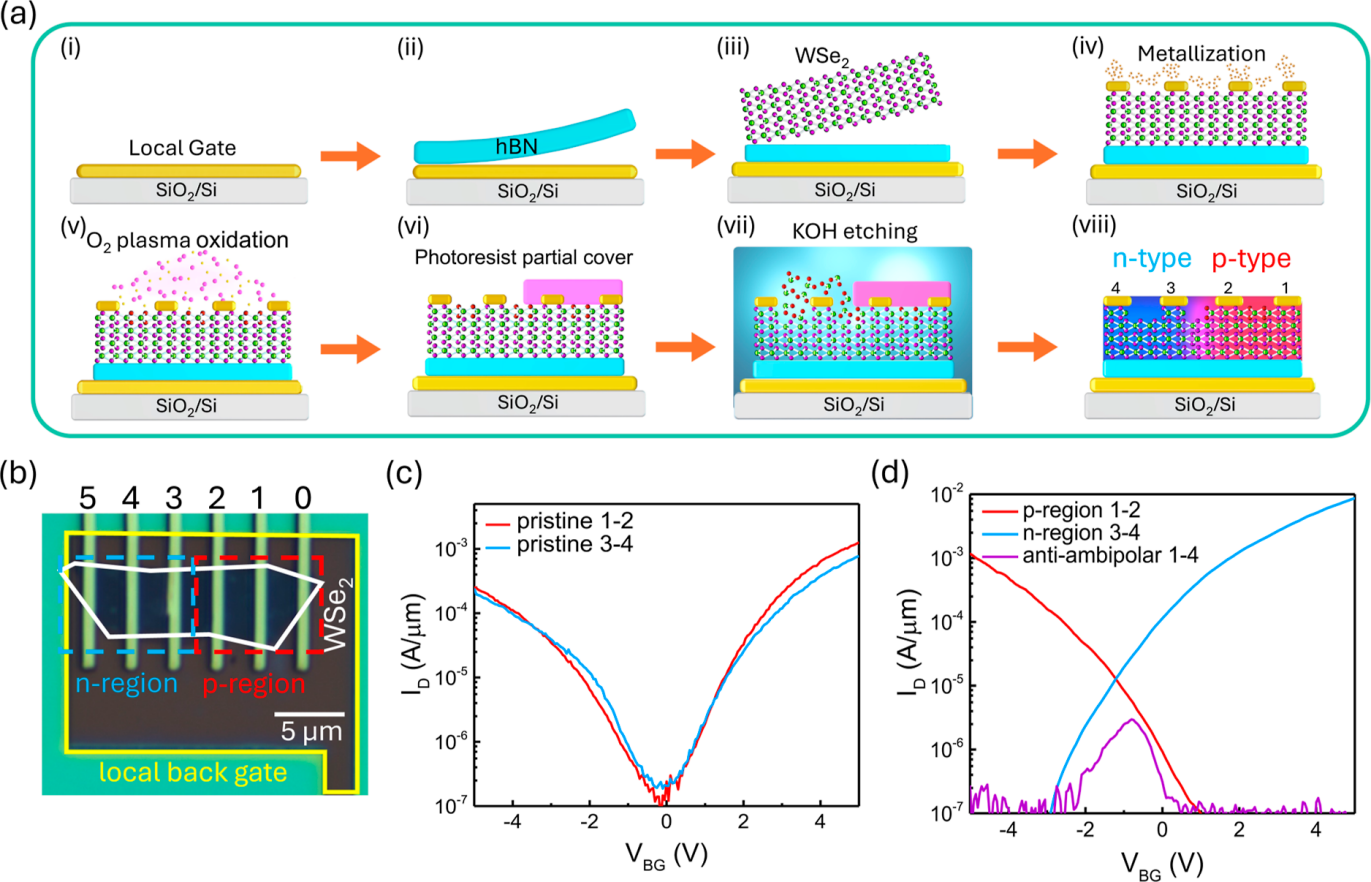
Fabrication process of the oxidized-pristine WSe_2_ lateral junction. (a) Fabrication process for the WSe_2_ p–n junction. (b) Optical microscope image of sample A, n- and p-regions are outlined in blue and red, respectively. The dark region corresponds to the bottom local back gate, covered by an hBN dielectric layer. (c) Electrical transfer characteristics of the pristine behavior, for contacts 1 and 2 (red trace) and contacts 3 and 4 (blue trace). (d) Corresponding transfer characteristics following O_2_ plasma treatment and KOH etching, showing p-type behavior (contacts 1–2) n-type behavior (contacts 3–4) and antiambipolar behavior (contacts 1–4). *V*_D_ = 1 V for panels (c) and (d).

To evaluate the electrical performance of this monolithic WSe_2_-based system for functional devices, FETs fabricated on the n- and p-regions in a single WSe_2_ flake are connected in an inverter circuit, as shown in Figure 3(a), inset. Voltage transfer characteristics and gain, defined from the slope |d*V*_out_/d*V*_in_|, are shown in Figure 3(a) for supply voltages *V*_D_ = 0.5 to 2 V. The corresponding power consumption is calculated as *P* = *V*_D_ × *I*_D_, where *I*_D_ is the static current through the inverter. The peak static power for this device is plotted in Figure 3(b), in comparison to various TMD-based inverters previously reported,^19,22,52–57^ further details of these devices are provided in comparison Table S1, Supporting Information. Our data are, to our knowledge, the lowest reported for TMD-based inverters at these supply voltages.^54,58^ The corresponding plot of power as a function of *V*_D_ for our device is given in Figure 3(c), where the peak static power consumption is 1.6 pW and 13.6 pW at *V*_D_ = 0.5 and 1 V, respectively.

**Figure 3:**
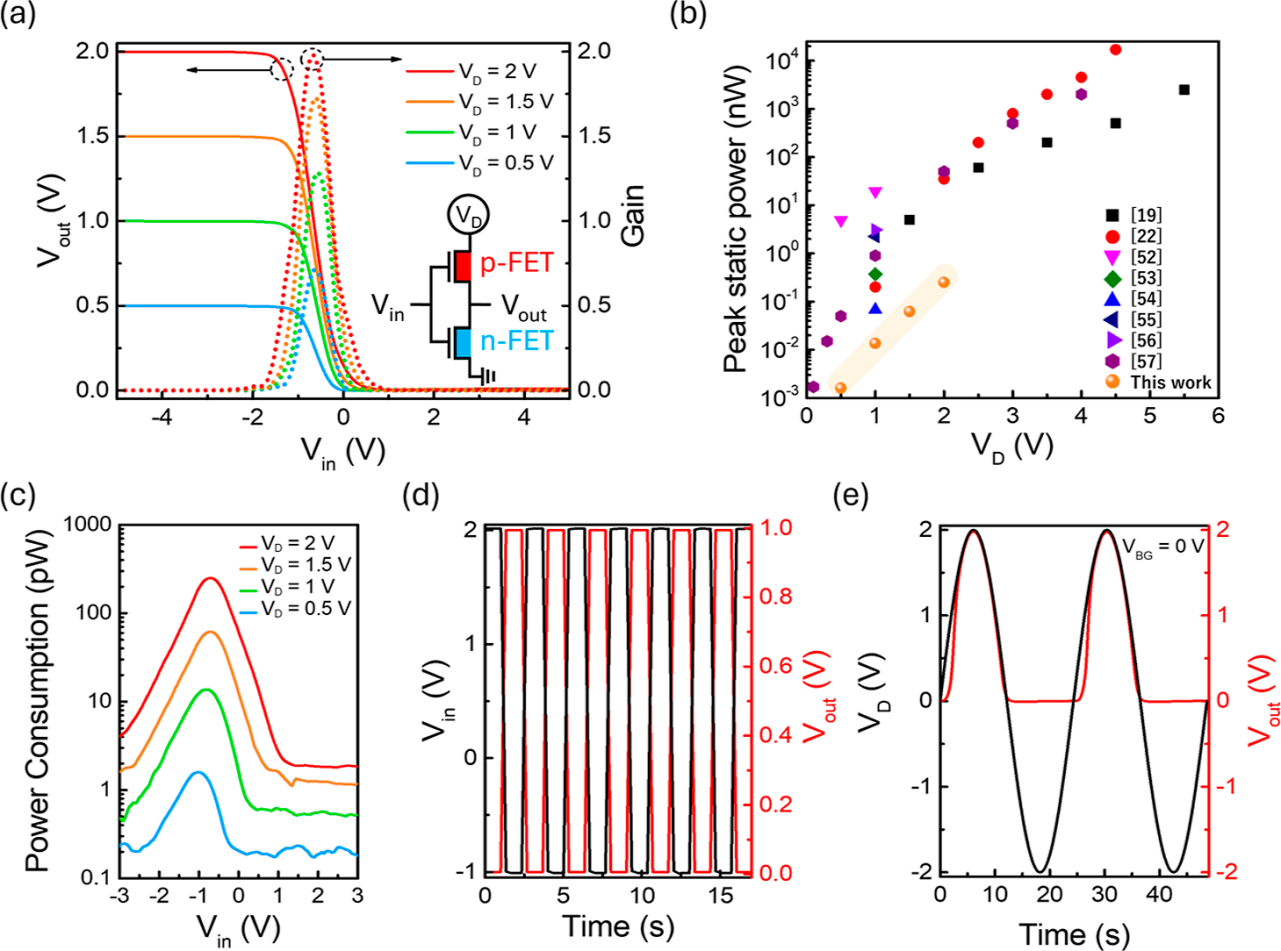
Electrical performance of the WSe_2_ inverter. (a) Inverter characteristics (*V*_in_ – *V*_out_) and voltage gain (|d*V*_out_/d*V*_in_|) for the circuit arrangement shown in the inset. (b) Comparison of peak static power consumption in reported 2D material-based inverters. (c) Corresponding inverter power consumption (*V*_D_ × *I*_D_) for *V*_D_ from 0.5 to 2 V. (d) Time-dependent response of *V*_out_ (red trace) for input pulse *V*_in_ (black). (e) Half-wave rectification behavior for a sinusoidal input.

Power dissipation is a critical design parameter affecting the feasibility of digital circuits,^59^ and minimizing power consumption is a central requirement for logic technologies.^60^ To achieve low-power inverters, both the supply voltage and the static current must be reduced.^61^ Since power and gain often scale together, voltage gain need only exceed unity to ensure logic-level restoration,^59^ and operation at modest gain values above unity is generally sufficient for energy-efficient circuit design.^62^ In this context, the oxide-based WSe_2_ inverter demonstrates strong potential for low-power logic by combining adequate gain exceeding unity at *V*_D_ = 1 V, for functional operation with ultralow power dissipation. Additionally, the inverter achieves a switching voltage between −1 and 0 V,^14,63^ Supporting Information Figure S4, reflecting the asymmetric characteristics of the WSe_2_ FETs with and without an oxide layer. A total of five inverter devices were fabricated. The corresponding gain and power consumption statistics are provided in Supporting Information Figure S5, showing average power consumptions of 1.7 pW and 11.1 pW at *V*_D_ = 0.5 and 1 V, respectively, and average gain values of 0.6 and 1.1 under the same bias conditions, respectively.

For completeness, the dynamic response of the inverter to a low-frequency square-wave input for *V*_D_ = 1 V is shown in Figure 3(d), confirming the inverted output. Half-wave rectification typical of a p–n junction diode is also observed by applying a low-frequency sine wave at *V*_D_ ^64^ (Figure 3(e)), with *V*_in_ = *V*_BG_ = 0 V, where the p–n junction is forward-biased during the positive half-cycle and reverse-biased during the negative half-cycle.

### Photoresponse

Beyond low-power logic, the built-in electric field of the oxidized/oxide-removed WSe_2_ junction enables self-powered photodetection in the photovoltaic regime. This regime has inherent advantages for fast response and low-noise operation, since carrier separation is driven by the built-in electric field of the p–n junction without the need for an external bias.^65,66^ Operating in this regime, the p–n junction shows linear current-power dependence, symmetric rise and fall times, fast temporal response, and key performance metrics for robust and reliable photodetection^65–69^ and is inherently suitable for low-power optoelectronic integration.

The photoresponse of the WSe_2_ p–n junction is measured under illumination with a 685 nm laser (photon energy 1.81 eV), exceeding the optical transition energy of three-to four-layer WSe_2_ (∼1.6 eV).^70^ The photoexcited *I*–*V* characteristics are shown in Figure 4(a), for *V*_BG_ = 0. Measurable photocurrent is observed even for very small laser excitation (50 nW), demonstrating high sensitivity along with an extremely low dark current of ∼250 fA under self-powered operation *V*_D_ = *V*_BG_ = 0 V (Supporting Information Figure S6). The laser spot is focused on the central region of the p–n junction, as indicated in Figure 4(b) and (c), and the energy band diagram for the illuminated state is shown in Figure 4(d). The relationship between photocurrent and laser power, Figure 4(e), can be described by a power-law dependence of the form *I*_ph_ = *a*·*P* ^α^, where *I*_ph_ is the photocurrent, *P* is the laser power, and α is the power-law exponent. Fitting yields α ≈1, indicate low recombination loss and low trap density,^65,71–73^ demonstrating that the oxide does not lead to significant charge trapping.

**Figure 4:**
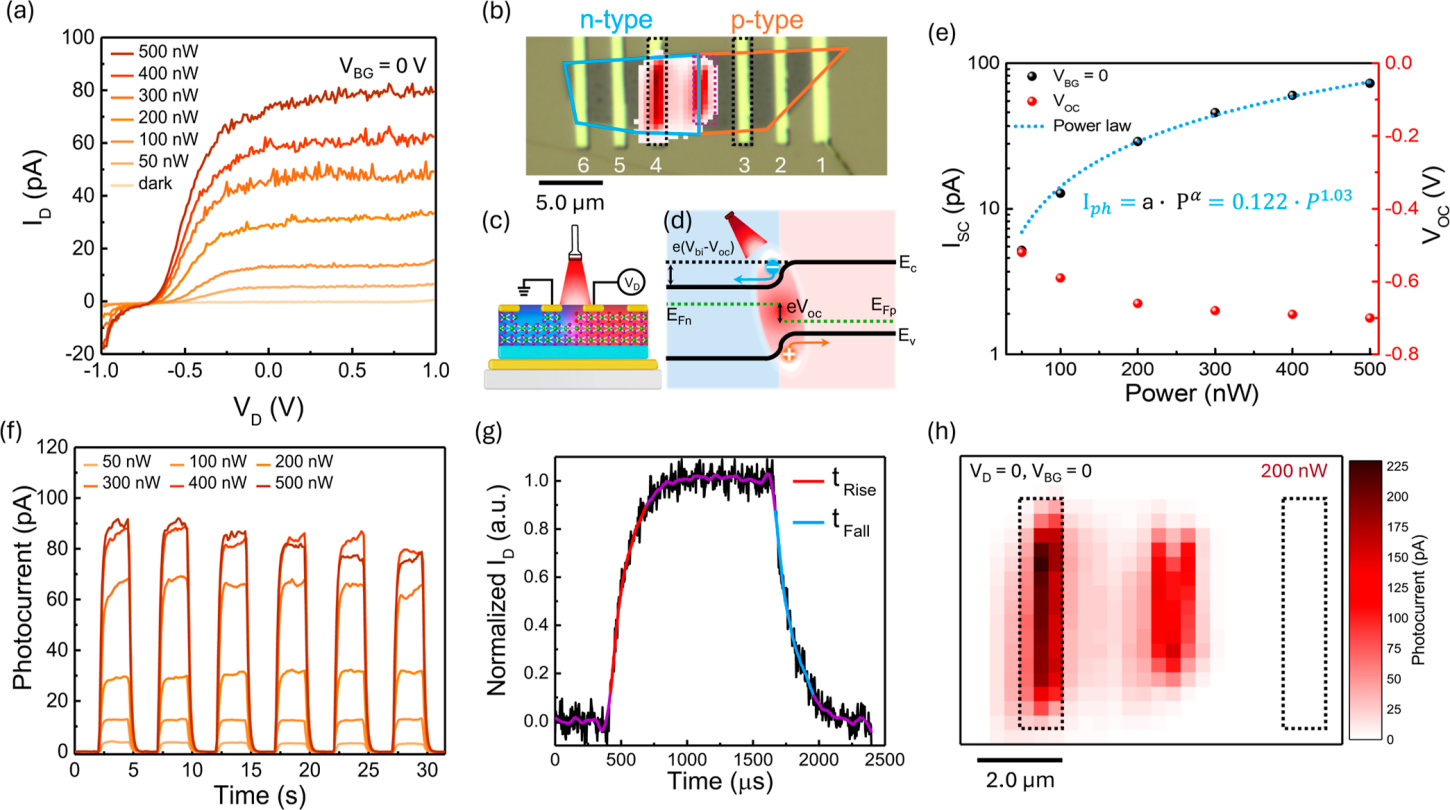
Photodetector characteristics of the WSe_2_ lateral p–n junction. (a) Current–voltage characteristics (*I*_D_ – *V*_D_) across the p–n junction (measured between contacts 3 and 4) under varying laser power at *V*_BG_ = 0 V. (b) Optical microscope image of sample B, the WSe_2_ p–n junction device, and n- and p-regions are outlined. (c) Schematic illustration of the photocurrent measurement setup. (d) Band diagram illustration of the WSe_2_ p–n junction under laser illumination. (e) Short-circuit current (*I*_SC_) and open-circuit voltage (*V*_OC_) as a function of laser power. (f) Time-dependent response of photocurrent for laser pulse with powers corresponding to those in panel (a). (g) Rise and fall time measurement of the photocurrent response. (h) Photocurrent mapping of the WSe_2_ flake at *V*_D_ = *V*_BG_ = 0 V, for 200 nW laser power. The black dashed lines indicate the position of contact electrodes.

The performance of the WSe_2_ p–n junction photodetector can be quantified through responsivity (*R*), external quantum efficiency (EQE), and specific detectivity (D*). The responsivity, defined as *R* = (*I*_ph_ – *I*_dark_)/*P*_in_, where *I*_ph_ is the photocurrent, *I*_dark_ is the dark current, and *P*_in_ is the incident laser power,^74^ is estimated to be in the range of 0.2 to 0.77 mA/W under 200 nW excitation in the self-powered regime. The estimate reflects spatial variation in the photoresponse, evidenced by the current mapping in Figure 4(h). The responsivity can be tuned by gate voltage and applied bias, increasing to ∼1.75 mA/W at *V*_D_ = *V*_BG_ = −1 V, Supporting Information Figure S6. The corresponding EQE, defined from the ratio of generated charge carriers to incident photons as EQE = *hcR*/λ*q*, where *h* is the Planck constant, gives EQE = 0.14% (self-powered) and 0.32% (biased at *V*_D_ = *V*_BG_ = −1 V). The photodetector performance is favorably competitive with previous studies of few-layer homogeneous WSe_2_ p–n junctions, where maximum responsivity *R* = 0.7 mA/W and EQE = 0.1% were achieved under biased operation for an electrostatically defined few-layer WSe_2_ p–n junction.^23^ The present device achieves comparable responsivity under self-powered operation while simultaneously exhibiting significantly faster temporal response. So far, research tends to focus on thick, multilayer flakes where greater light absorption and higher carrier density naturally lead to higher responsivities^15,34,74^ but can show slow response times, in the millisecond range,^74^ several orders of magnitude higher than our few-layer devices. The temporal photoresponse for our device under pulsed illumination, Figure 4(f), shows stable and reproducible on–off switching (50–500 nW) at *V*_D_ = *V*_BG_ = 0 V. Representative rise and fall times, Figure 4(g), are 307 and 315 μs, respectively. The symmetric, fast, and repeatable response indicates minimal charge trapping and the absence of photogating effects. These response times are competitive with the fastest homogeneous WSe_2_-based p–n junctions, where rise and fall times of 200 and 16 μs, respectively, have been reported using combined chemical and electrostatic doping.^15^ Comparison with other TMD-based photodetectors is given in the Supporting Information, Tables S2 and S3.

For completeness, the specific detectivity^74–76^ $D^{*}=R\sqrt{A}/\sqrt{2qI_{dark}}$ is 1.6 × 10^9^ and 2.6 × 10^9^ Jones for self-powered and biased conditions, respectively. Here, the effective device area *A*, is taken to be the entire channel area, where *L* = 6 μm and *W* = 6 μm. We note that localized laser spot excitation likely leads to an underestimation of responsivity and related metrics, as the illuminated area is significantly smaller than the effective p–n junction area, corresponding to an estimated ∼6-fold difference (Supporting Information Figure S7).

Finally, Figure 4(h) shows spatial mapping of the photocurrent for *V*_D_ = *V*_BG_ = 0 V and 200 nW laser power, showing clear charge separation at the p–n junction. The black dashed lines mark contact positions. A measurable photocurrent is also observed at the metal–n-semiconductor contact, consistent with charge separation from Schottky contact formation. Back-gate-dependent photocurrent maps are shown in Supporting Information, Figure S7. In summary, the WSe_2_ lateral p–n junction functions very well as a conventional photovoltaic photodiode, offering a robust, fast, and linearly responsive, bias-free photodetection in two-dimensional materials. Finally, the stability of the oxide layer is an important consideration for practical device applications. Previous studies have shown that oxidized WSe_2_ regions adjacent to contacts exhibit stable electrical characteristics under ambient conditions over extended periods.^18^ However, the dependence of long-term stability on the size and geometry of the oxidized region remains a topic for future investigation.

## Conclusions

We performed nanoscale mapping of the bias-dependent local capacitance across a WSe_2_ lateral junction precisely defined by oxidation and selective oxide removal, using a modified bb-EFM technique optimized for 2D materials. A clear contrast is observed between the oxidized regions and those where the oxide is selectively removed, confirming p-type characteristics in the oxide-covered area and n-type behavior in the pristine region, with a work function difference of ∼120 meV. The broadband excitation and nondestructive nature of bb-EFM are suitable for characterizing carrier concentrations over a wide range while preserving the structural integrity of delicate van der Waals systems, thereby establishing a powerful surface-microscopy-based approach for quantitatively probing carrier contrast in 2D semiconductor materials, without the influence of electrical contacts. Electrical measurements confirm the p- and n-type characteristics of the oxidized and oxide-removed regions, respectively, and integrating FETs fabricated on the n- and p-regions in an inverter circuit shows record-low peak static power consumption for TMD-based inverters. The p–n junction functions as an efficient self-powered photodetector operation in the photovoltaic region, with linear current-power dependence and ultrafast submillisecond temporal response. Together, these results demonstrate WSe_2_ with controllable oxidation as a promising platform for low-power logic and optoelectronic devices based on 2D semiconductors.

## Methods

### Device Fabrication

The WSe_2_ crystals were purchased from HQ Graphene and mechanically exfoliated onto a highly doped 285 nm SiO_2_/Si substrate. Hexagonal boron nitride (hBN) crystals were also exfoliated onto SiO_2_/Si substrates. The hBN samples were annealed to remove surface residue by gradually heating to 300 °C over 1 h, maintaining for 3 h, and allowing to cool naturally. To fabricate devices for electrical measurements, locally defined back gates were patterned on separate 285 nm SiO_2_/Si substrates using electron beam (e-beam) lithography, followed by e-beam evaporation to deposit 10/30 nm Cr/PdAu. The hBN and WSe_2_ flakes were transferred onto the local gate using a polycarbonate–polydimethylsiloxane (PC–PDMS) dry transfer technique. Residual PC on the sample surfaces was removed using chloroform, acetone (ACE), and isopropanol (IPA). The source and drain electrode patterns were defined by e-beam lithography after spin-coating the device with a copolymer/PMMA A4 bilayer. Metal electrodes were deposited using e-beam evaporation (20/70 nm Cr/PdAu). The oxidation process was conducted using a reactive ion etching (RIE) system with low power (10 W) and a low flow rate (15 sccm O_2_) for 120 s at room temperature. Copolymer/PMMA A4 served as a protective mask during e-beam lithography to define specific regions, exposing selected areas of the WO_*x*_ layer. The exposed regions were then etched using a sequence of 1 M KOH (15–60 s), deionized (DI) water (60 s), and IPA, effectively removing the surface WO_*x*_. Finally, ACE and IPA were used to remove the residual photoresist.

Separate devices were made for surface microscopy measurement (KPFM and bb-EFM). A graphite flake is first transferred onto a substrate containing prepatterned contact electrodes for grounding. The WSe_2_ flake is then transferred onto the graphite flake using the same PC–PDMS transfer technique described above. The same entire surface oxidation and selective area oxide removal scheme (as described above) were used to define lateral p- and n-regions.

### KPFM and bb-EFM Measurements

The bb-EFM measurements were performed on a commercial AFM (Multimode 8, Bruker) with a Nanoscope V controller operated in tapping mode and equipped with a conductive tip holder for combined DC and AC excitation. Pt-/Ir-coated cantilevers (NCSTPt, Nanosensors) with a nominal spring constant of $∼7.4Nm^{-1}$ and resonance frequency *f*_0_ = 160 kHz were used. A function generator (Keysight 33521B) supplied the radio frequency excitation. An amplitude-modulated waveform was applied, consisting of a 1–5 GHz carrier frequency (*f*_car_) modulated at 2 kHz (*f*_mod_) with a power of 15 dBm, described by *V*_AM_(*t*) = *V*_AC_[1 + sin­(2π*f*_mod_ *t*)] sin­(2π*f*_car_ *t*). The capacitive force response was demodulated at *f*_0_ ±2 kHz using a lock-in amplifier (HF2LI, Zurich Instruments), yielding an enhanced signal-to-noise ratio. The voltage-dependent bb-EFM amplitude was calibrated using graphite electrodes as a reference.

Sideband KPFM measurements were performed on the same AFM platform in tapping mode, with the cantilever resonance tracked by a phase-locked loop at *f*_0_ ≈160 kHz. A 2 kHz AC modulation was applied between the tip and sample together with a DC feedback bias. The sideband signal at *f*_0_ ±2 kHz was detected using the HF2LI lock-in amplifier and served as the KPFM feedback source. In closed-loop operation, the DC bias was adjusted to null the sideband response, producing quantitative maps of the local contact potential difference.

### Electrical Measurements

Electrical characteristics were measured using a Keysight B1500A semiconductor parameter analyzer at pressures between 10^–4^ and 10^–7^ hPa in a Lake Shore cryogenic probe station. Photocurrent measurements were performed using a Thorlabs MCLS 685 nm diode laser, with a Keithley 2636B and Moku:Go oscilloscope and function generator.

## Supplementary Material



## Notes

Photoluminescence calibration of WSe_2_ thickness; Raman and photoluminescence characterization of oxide formation; Schottky barrier extraction; switching voltage; average power consumption and gain; backgate-dependent photodetector measurements; gate-dependent photocurrent mapping; photodetector responsivity; and temporal response (PDF)
