# Supplementary material for: Ultra-Low Power CMOS Logic and Photodetection in WSe2 Semiconductors by Selective Area Plasma Doping
Source: ACS Appl Mater Interfaces. 2026 Jun 22;18(25):36187–96. doi: 10.1021/acsami.6c04479 (PMC13339015; doi:10.1021/acsami.6c04479)
Supplement: Supplementary file 1 [file am6c04479_si_001.pdf]

Supporting Information: Ultra-Low Power CMOS logic and Photodetection in WSe_2 Semiconductors by Selective Area Plasma Doping

Jheng-Jie Lin[1], He-Yu Chen[1], Sheng-Zhu Ho[1], Xiaofan Lin[4], Greg Chu[4], Faris Abualnaja[4], Zhen-You Lin[1], Yu-Chiang Hsieh[1], Kuo-En Chang[1], Chung-Lin Wu[1], Kenji Watanabe[5], Takashi Taniguchi[6], Yi-Chun Chen[1,2], Tse-Ming Chen[1, 2], Jack A. Alexander-Webber*[4,7], and Luke W. Smith*[1, 2, 3]

[1]Department of Physics, National Cheng Kung University, Tainan 701, Taiwan
[2]Center for Quantum Frontiers of Research & Technology (QFort), National Cheng Kung University, Tainan 701, Taiwan
[3]Academy of Innovative Semiconductor and Sustainable Manufacturing, National Cheng Kung University, Tainan 701, Taiwan
[4]Electrical Engineering Division, Department of Engineering, University of Cambridge, Cambridge CB3 0FA, U.K.
[5]Research Center for Electronic and Optical Materials, National Institute for Materials Science, 1-1 Namiki, Tsukuba 305-0044, Japan
[6]Research Center for Materials Nanoarchitectonics, National Institute for Materials Science, 1-1 Namiki, Tsukuba 305-0044, Japan
[7]Department of Materials, Loughborough University, Loughborough, LE11 3TU, U.K.

*Email: jaa59@cam.ac.uk
lukesmith@phys.ncku.edu.tw

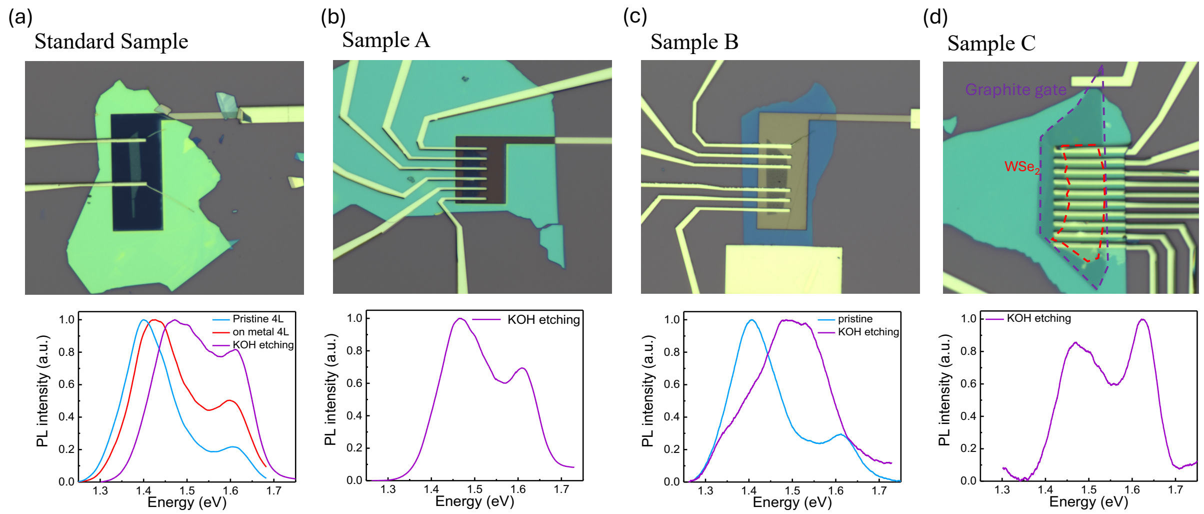

Figure S1: (a) Optical micrograph (OM) and photoluminescence (PL) spectra of the standard WSe_2 sample under different conditions: pristine on SiO_2, on a local gate/hBN, and after KOH etching. (b) PL spectrum of Sample A after KOH etching, showing a thickness reduction from four to three layers (used for electrical measurements). (c) PL spectra of Sample B before and after KOH etching, confirming the thickness change from four to three layers (used for photodetector measurements). (d) PL spectrum of Sample C after KOH etching, with the layer number reduced from four to three (used for photocurrent response measurements).

Photoluminescence calibration of WSe_2 thickness

Four-layer WSe_2 is used for all devices in this work. Photoluminescence (PL) is first measured on a standard sample, the optical micrograph and PL measurements are given in Figure S1(a). The blue trace shows the PL measurement after exfoliation and before transfer to the back-gate structure, confirming four-layer WSe_2 [1]. After transfer to the metal back gate/hBN structure, a blue shift is observed in the PL spectra (red trace). A low-power O_2 plasma treatment is employed to induce controlled surface oxidation. The purple trace shows the PL spectra measured in the etched region after oxidation and KOH etching, and exhibits a transition from four-layer to three-layer characteristics [1], consistent with the removal of a single oxidized surface layer. This is consistent with prior reports [1–8] of self-limited oxide formation confined to the uppermost surface of WSe_2, and is further discussed in the following section in relation to Figure S2. Inverter measurements were performed using sample A, while optical measurements were predominantly performed using sample B, with rise- and fall-time measurements performed using sample C. The optical microscope images and PL spectra for samples (a), (b), and (c) are shown in Figure S1(b), (c), and (d), respectively. Sample C is fabricated with a graphite rather than metal back-gate.

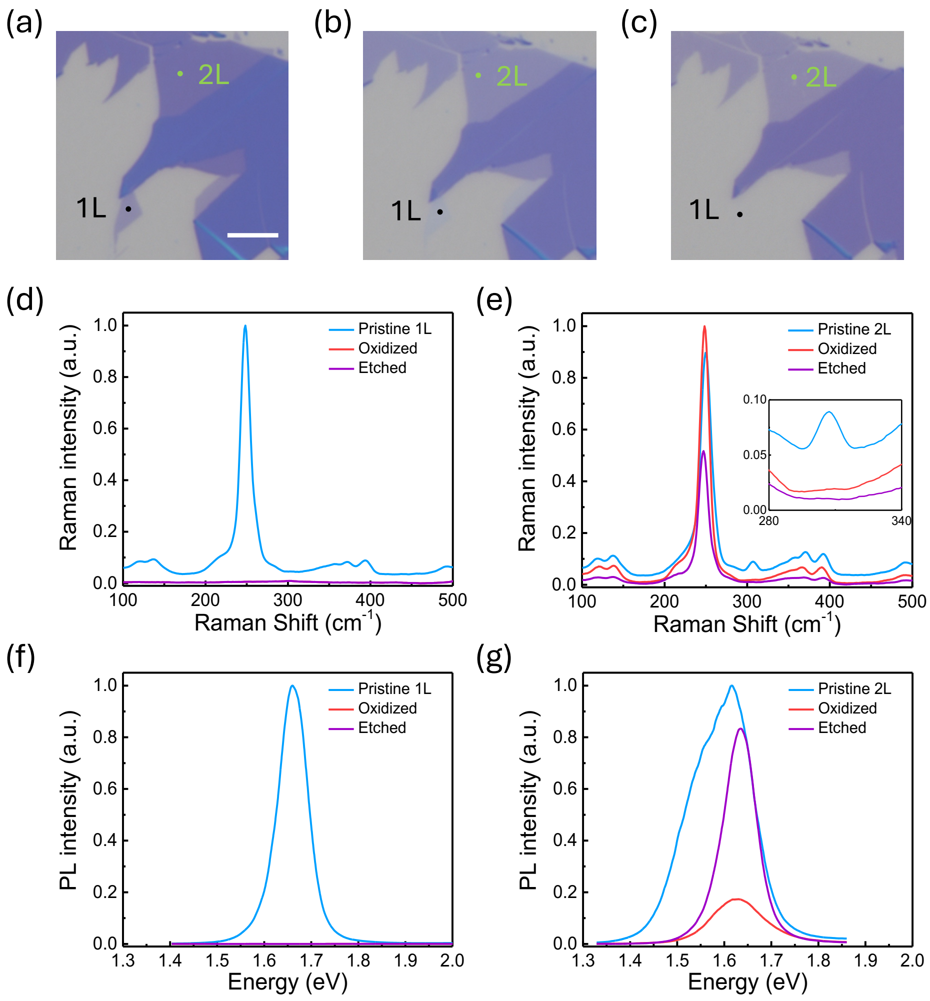

Figure S2: (a), (b), (c) Optical micrographs of pristine, oxidized, and etched WSe_2, respectively. The scale bar in (a) corresponds to 5 μm, and is the same for all optical micographs. (d) and (e) Raman spectra of 1L and 2L WSe_2, respectively. Data are shown for pristine, oxidized, and etched conditions. (f) and (g) Corresponding PL spectra for 1L and 2L WSe_2, respectively, for pristine, oxidized, and etched conditions.

Raman and photoluminescence characterization of oxide formation

Figure S2(a) shows an optical micrograph of pristine WSe_2 containing both monolayer (1L) and bilayer (2L) regions (lablelled). Figure S2(b) shows the sample after O_2 plasma oxidation. The color contrast of the 2L region becomes noticeably weaker and resembles that of pristine 1L WSe_2, while the original 1L region becomes nearly invisible.[1,9] Figure S2(c) shows the sample after subsequent KOH etching, where the original 1L region completely disappears. Figure S2(d) shows the Raman spectra of pristine monolayer (1L) WSe_2 before and after oxidation and KOH etching. After oxidation and etching, the combined $A_{1g} + E_{2g}^1$ peak $\sim$248 cm^{-1} disappears, consistent with transformation of the WSe_2 to oxide[1] and its subsequent removal after etching[9,10]. Figure S2(e) presents the Raman spectra of pristine bilayer (2L) WSe_2 before and after oxidation and etching. Following oxidation and KOH treatment, the B_{2g}^1 peak $\sim$307 cm^{-1} disappears[1,10], and the resulting Raman spectrum becomes consistent with that of pristine monolayer WSe_2. The PL spectra shown in Figure S2(f) further support this behavior. After oxidation and KOH etching of pristine monolayer

WSe_2, the original PL peak at 1.66 eV disappears, indicating removal of the oxidized monolayer region. In addition, Figure S2(g) shows that, after oxidation and etching of pristine bilayer WSe_2, the characteristic bilayer PL peaks at 1.55 and 1.61 eV become a single peak at 1.66 eV, consistent with the PL response of pristine monolayer WSe_2. These results support selective oxidation primarily of the uppermost WSe_2 layer by the low-power O_2 plasma treatment,[1–8] although there may be preferential oxidation at the edges of the flake[11,12].

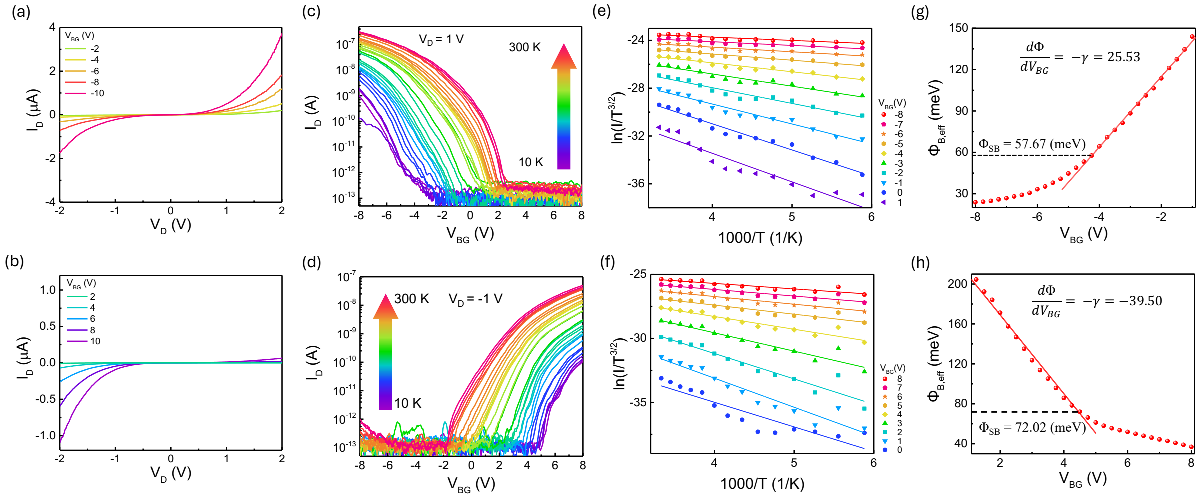

Figure S3: (a), (b) Current-voltage characteristics (I_D-V_D), for p- and n-regions, respectively, sample A. (c), (d) Corresponding electrical transfer characteristics (I_D–V_{BG}), measured over the temperature range from 10 K to 300 K. (e, f) Richardson plots $\ln(I_D/T^{1.5})$ as a function of $1000/T$, extracted from panels (c) and (d), respectively. (g), (h) Effective Schottky barrier height (SBH) as a function of gate voltage, obtained from (e) and (f), respectively.

Schottky barrier extraction

Schottky barrier values are extracted from temperature dependent measurements as shown in Fig. S3. The non-linear and asymmetric $I - V$ sweeps, Figure S3(a) and (b), confirm the presence of a Schottky barrier for the p- and n-regions, measured using contacts 1-2 and 3-4, respectively, sample A. Temperature-dependent transfer characteristics for the p- and n-regions are shown in Figure S3(c) and (d), respectively, from $T = 10$ to 300 K. The corresponding Richardson plots $\ln(I_D/T^{1.5})$ versus $1000/T$ are shown in Figure S3(e) and (f), respectively, for fixed V_{BG} values. The effective Schottky barrier height $\Phi_{B,\mathrm{eff}}$ can be extracted using the thermionic emission equation[13,14]

$$\Phi_{B,\mathrm{eff}}(V_{BG}) = \frac{k_B}{q}\left[\frac{\Delta \ln\left(\frac{I_D(V_{BG})}{T^{3/2}}\right)}{\Delta\left(T^{-1}\right)}\right] ,$$

where k_B is the Boltzmann constant, q is the carrier charge, and T is the temperature. The effective Schottky barrier $\Phi_{B,\mathrm{eff}}$ can therefore be estimated from the gradient of Richardson plots. Figure S3(g) and (h) show $\Phi_{B,\mathrm{eff}}$ as a function of V_{BG} for p- and n-regions, respectively, showing the dependence of the effective Schottky barrier on V_{BG}. For n-type FETs, as V_{BG} increases from the off-state, charge transport is dominated by thermionic emission due to the relatively large barrier height. The effective barrier height is gradually reduced by further increasing V_{BG}, and the flat-band condition can be identified from the inflection point in Figure S3(g) and (h). This transition marks a change in the dominant transport mechanism and provides an estimate of the SBH[15–17].

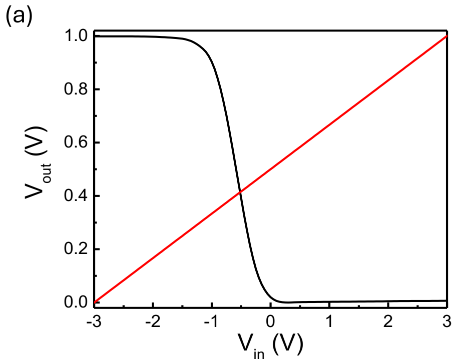

Figure S4: (a) Inverter switching voltage for $V_{\mathrm{D}} = 1$ V.

Switching voltage

The inverter switching voltage (V_{M}) is estimated following Refs.[18,19] using the linear equation $V_{\mathrm{out}} = (V_{\mathrm{D-range}}/V_{\mathrm{in-range}}) \times (V_{\mathrm{in}} + 3)$, where $V_{\mathrm{D-range}}$ and $V_{\mathrm{in-range}}$ are the supply and input voltage ranges, respectively. The offset term $(V_{\mathrm{in}} + 3)$ accounts for the offset between input and output voltage ranges, *i.e.*, $V_{\mathrm{in-range}}$ between ± 3 and V_{out} from 0 to 1. The switching voltage defines the transition between zero and one states. Figure S4(a) shows the inverter voltage transfer curve for $V_{\mathrm{D}} = 1$ V (black trace) and linear equation defined above (red trace). The switching voltage is given by the intercept between the two. Here we estimate $V_{\mathrm{M}} = -0.5$ V.

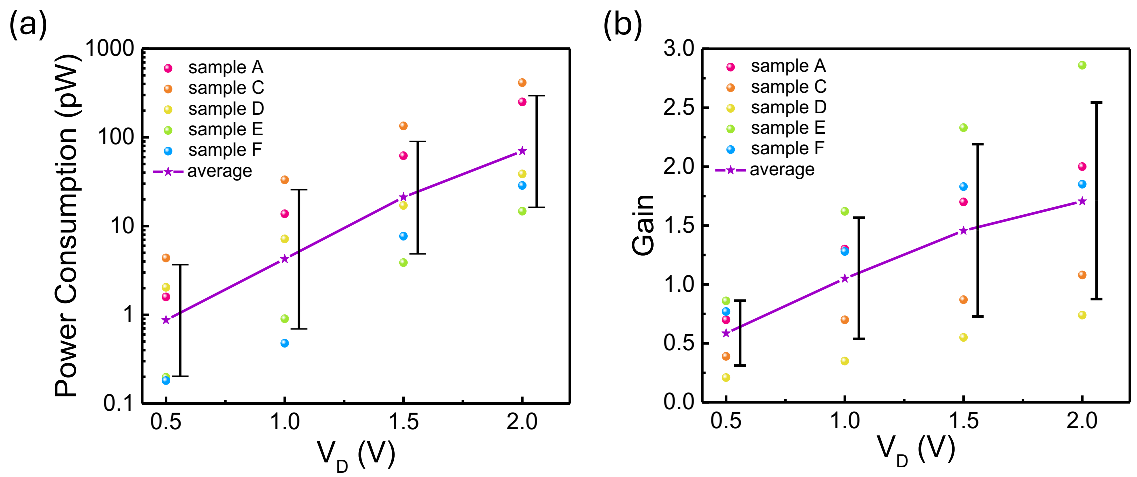

Figure S5: (a) and (b) Average power consumption and voltage gain, respectively, for five different samples as a function of V_{D}.

Average power consumption and gain

Figure S5(a) shows the power consumption plotted on a logarithmic scale, including standard deviation error bars. Since the power values span multiple orders of magnitude for different V_{D}, the statistical analysis was performed in logarithmic space. The raw power values were transformed using $\log_{10}$, after which the mean and standard deviation were calculated. The plotted values and error bars were then converted back to linear scale as 10^{Mean}, $10^{(\mathrm{Mean+SD})}$, and $10^{(\mathrm{Mean-SD})}$, respectively. Figure S5(b) shows the corresponding average gain values, including standard deviation error bars, with average values of 0.6 and 1.1 at $V_{\mathrm{D}} = 0.5$ and 1 V, respectively. The data presented in the main manuscript correspond to Sample A, which is representative of the device ensemble.

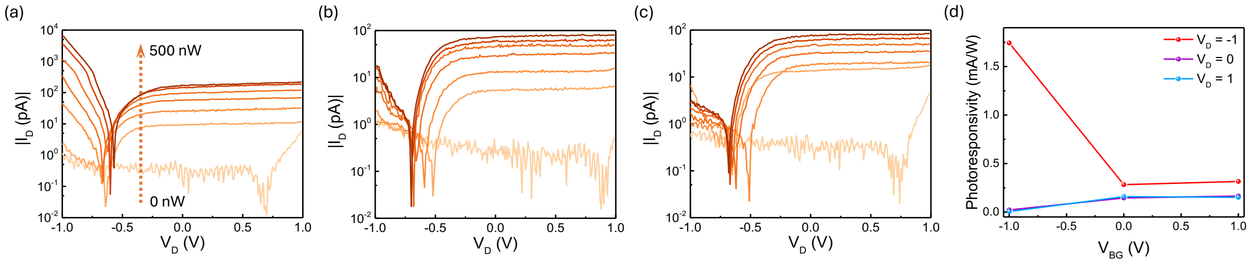

Figure S6: (a), (b), and (c) Current-voltage characteristics ($|I_D|$-V_D) across the pn junction at $V_{BG} = -1$, 0, and 1 V, for laser powers from dark, 50, 100, 200, 300, 400, to 500 nW. The absolute value of current is plotted. (d) Responsivity of the photodiode as a function of both V_D and V_{BG} for 200 nW laser power.

Backgate-dependent photodetector measurements

Figure S6(a), (b), and (c) show the gate dependence of the photocurrent at $V_{BG} = -1$, 0, and 1 V, respectively. Under self-powered conditions, $V_D = V_{BG} = 0$ V, the dark current is $\sim$ 250 fA. These measurements are performed with the laser spot positioned nominally at the center of the junction to perform bias- and gate-dependent characterization. The corresponding responsivities are plotted in Figure S6(d), calculated using $R = (I_{ph} - I_{dark})/P_{in}$ for a laser power of 200 nW, demonstrating gate- and bias-dependent tunability. As shown in the spatial mapping results, Figure 4(h) (main manuscript), and Figure S7, there is some variation of photoresponse with position. The maximum responsivity of 0.77 mA/W reported in the manuscript corresponds to the highest photocurrent observed in the mapping measurements for self-powered operation, $V_D = V_{BG} = 0$ V.

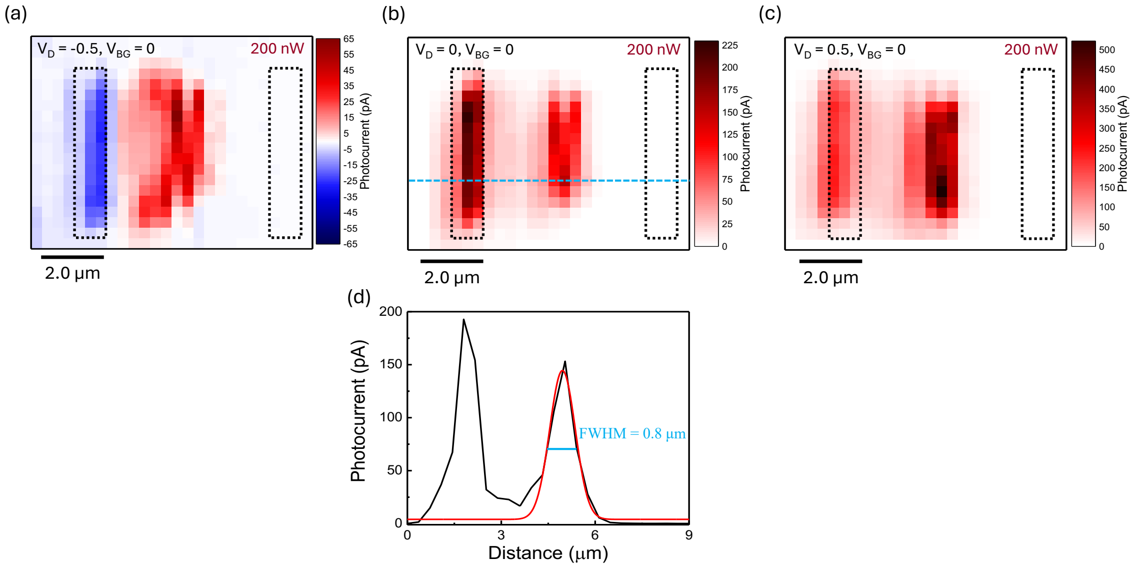

Figure S7: (a), (b), and (c) Photocurrent mapping images of the WSe_2 pn junction at V_D= -0.5 V, 0 V, and -0.5 V, respectively, with V_{BG} = 0 V, laser power = 200 nW. (d) Photocurrent profile corresponding to the blue dashed line in (b), indicating the full width at half maximum (FWHM) of the photocurrent distribution.

Gate dependent photocurrent mapping

Spatial photocurrent mapping of the WSe_2 flake for $V_D = -0.5$, 0 and 0.5 V is shown in Figure S7 (a), (b), and (c), respectively, for $V_{BG} = 0$ V. Metal contact positions are marked by the black dashed outlines. The largest photocurrent is observed for forward bias, $V_D = 0.5$ V. To estimate the width of the pn-junction depletion region, the photocurrent is plotted as a function of horizontal position, Figure S7(d) for $V_D = V_{BG} = 0$ V, corresponding to the blue line in Figure S7(b). The full width at half maximum is extracted to be ~ 0.8 μm. A ~ 1 μm diameter laser spot is used for all photodetector measurements, rather than light incident on the entire channel. Comparing the geometric area mismatch between the spot area and pn junction region suggests a possible $\sim$ 6-fold difference, where the spot area is estimated as πr^2 with $r \sim 0.5$ μm and the pn junction area as $W = 6$ μm and $L = 0.8$ μm. This likely leads to significant underestimate of the responsivity and other performance metrics.

Photodetector responsivity and temporal response

We estimate photodetector responsivity R and external quantum efficiency EQE of 0.77 mA/W and 0.14%, respectively, for self powered conditions, as detailed in the main manuscript. These values can be tuned by the back gate, increasing to $\sim$1.75 mA/W and 0.32% when biased at $V_D = V_{BG} = -1$ V. These results are favorably competitive with previous studies of few layer homogeneous WSe_2 pn junction based photodetectors, where maximum values of R = 0.7 mA/W and EQE = 0.1% where achieved using an electrostatically-doped device biased at $V_D = -1$ V[20]. Naturally, higher responsitivity can be achieved for thicker material flakes due to the increased fraction of light absorbed, higher carrier density, and consequently increased photocurrent[21]. However, the enhanced responsivity can be at the expense of slower response time[22,23]. A comparision of photodetector performance metrics for TMD-based devices is given in Table S2. Corresponding response times, where available, are given in Table S3. For example, values of R = 2000 mA/W and EQE = 420% at $V_D = 1$ V, $V_G = 0$ V, have been achieved for relatively thick multi-layer monolithic WSe_2 junctions. However, rise and fall times of order tens and thousands of ms, respectively, were measured[14], several orders of magnitude higher than our few-layer devices. Supplementary information of Ref.[14] also reports R from < 1 to ~ 20 mA/W for thinner, 6 nm WSe_2 ($\sim$ 8 layers), much closer to values reported for our three layer devices.

To further address the thickness-dependence of light absorbed, we apply the Beer-Lambert law, where the absorbed fraction is given by $A = 1 - e^{-\alpha d}$, where d is the layer thickness, approximating 0.7 nm per layer, and α is the absorption coefficient given by $\alpha = 4\pi k/\lambda$, where k is the extinction coefficient and λ is the laser wavelength. For three-layer WSe_2, $k \approx 0.54$[24,25], giving $A \sim 20\%$. For comparison, $k \approx 0.87$ for five-layer WSe_2[24,26], giving $A \sim 55\%$. The significant increase in absorption with the addition of only two layers (three to five) helps illustrate the thickness dependence of device performance.

We note that operation in the photovoltaic regime contrasts with phototransistors that rely on photogating effects, which typically require an applied bias and are associated with charge trapping, leading to large dark currents, slow temporal response, and device instability. For devices such as ours, the absence of an applied gate or drain voltage, as well as carrier trapping, generally results in lower responsivity but faster respsonse compared to photogating-dominated devices[22,23].

The rise and fall times of 307 and 315 μs, respectively, measured in our device are fast and comparable to reported response times for WSe_2-based pn junction photodetectors. For example, rise and fall times of 200 and 16 μs have been reported for a homogeneous WSe_2 pn junction formed using a combination of chemical and electrostatic doping, measured at zero drain bias and with a large back-gate voltage applied[27]. Even faster rise and fall times of 264 and 552 ns have been reported for a p–i–n structure combining plasma treatment (Ar at the n-contact, O_2 at the p-contact), with optimized work-function metals (Pd/Au for p-contacts, and Ti/Au for n-contacts), operated at zero bias[28]. Nanosecond-scale response times are exceptionally rare among TMD-based photodetectors, here relying on more elaborate material and contact engineering. A comparison of response times for TMD pn-junction based photodetectors are given in Table S3.

Table S1: Gain and power consumption comparison table for TMD-based inverters.

**Material structure**	**Polarity control**	**V_D**	**Peak static power consumption**	**Gain**	**Measurement environment**	**Refs.**
WSe_2/n^+-Si and MoS_2/p^+-Si	n^+-Si / p^+-Si	0.5 V / 1 V	4.89 nW / 19.4 nW	16.6/19.6	ambient air room temperature	29
CVD-grown p-type $MoTe_2$ n-type MoS_2	Intrinsic property	1 V	0.37 nW	1.5 / 2.3	Dark ambient	30
WSe_2 / MoS_2 negative capacitance	Negative capacitance gate stack, ALD-induced doping	1 V / 2 V	68 pW / 68 pW	11.6	$< 10^{-5}$ bar	31
WSe_2/SiO_2 WSe_2/hBN pn homojunction	UV LED-induced doping	1 V	2.25 nW	0.06	—	32
n-type MoS_2 p-type WSe_2	Intrinsic property	1 V	3.1 nW	4	—	33
WSe_2 with K-doped n^+ contacts; pristine WSe_2	SCTD with potassium	1 V<br>2 V<br>3 V	—	3<br>7<br>13	—	34
SnO_x / WSe_2 $CuInS_2$ / WSe_2	surface charge transfer doping	3 V / 4V<br>5 V	—	55 / 140<br>176	—	35
vdW contact / WSe_2 Evaporated contact / WSe_2	SCTD Contact induced	1.5V / 2.5V<br>3.5V /4 .5V<br>5.5 V	—	30 / 125<br>170 / 270<br>340	room temperature in vacuum	36
Ni/Pt / WSe_2 Pd/Au / WSe_2	Contact induced	3 V	—	79	ambient air	37
In / WSe_2 Pt / WSe_2	Contact induced	4.5 V	10 μW	198	10^{-5} bar	38
Oxidized WSe_2 Pristine WSe_2	O_2 plasma doping and KOH etching	0.5 V / 1 V<br>1.5 V / 2 V	1.6 pW / 13.6 pW<br>62 pW / 251 pW	0.7 / 1.3<br>1.7 / 2	10^{-6} bar Dark	This work

Table S2: Comparison table for TMD-based photodetector devices.

**Material structure**	**Thickness**	**Measurement Conditions**	**Laser Power**	**Responsivity**	**EQE**	D^*(Jones)	**Refs.**
$MoS_2/WO_x/WSe_2$	6/2/5 nm	520 nm ($V_D = 0V, V_G = 0V$)	344 $\mu W/cm^2$	11.75 mA/W	2.8 %	2.8×10^{10}	39
		852 nm ($V_D = 0V, V_G = 0V$)	235 $\mu W/cm^2$	0.19 mA/W	0.028 %	4.6×10^8	
Oxidized WSe_2	multilayer	520 nm ($V_D = 1V, V_G = 0V$)	310 μW	250 mA/W	97 %	7.7×10^9	14
		852 nm ($V_D = 1V, V_G = 0V$)	75 μW	2000 mA/W	420 %	7.2×10^9	
Oxidized WSe_2	multilayer	532 nm (High gate bias)	84 nW	7.1×10^4 mA/W	228 %	3×10^3	40
$NbSe_2/MoS_2$	37.1/35.1 nm	660 nm ($V_D = 0V, V_G = 0V$)	5.2 mW/cm^2	455.3 mA/W	85.7 %	1.9×10^{12}	41
PEI doping WSe_2	90 nm	520 nm ($V_D = 0V, V_G = 40V$)	1.8 nW	80 mA/W	20 %	10^{11}	27
Gate tunable WSe_2	few layer	532 nm ($V_D = -1V$)	840 mW/cm^2	0.7 mA/W	0.1 %	—	20
Oxidized WSe_2	3.5 nm	685 nm ($V_D = 0V, V_G = 0V$)	200 nW	0.77 mA/W	0.14 %	1.6×10^9	This work

Table S3: Rise and fall time comparison of TMD-based photodetector devices.

Material structure	Thickness	Measurement Conditions	Rise Time	Fall Time	Refs.
$NbSe_2$ / MoS_2	37.1/35.1 nm	660 nm ($V_D = 0V$, $V_G = 0V$)	17 μs	18 μs	41
$MoS_2/WO_x/WSe_2$	6/2/5 nm	520 nm ($V_D = 0V$, $V_G = 0V$)	500 μs	842 μs	39
		852 nm ($V_D = 0V$, $V_G = 0V$)	793 μs	844 μs	
Oxidized WSe_2	multilayer	520 nm ($V_D = 1V$, $V_G = 0V$)	41.8 ms	2289.8 ms	14
		852 nm ($V_D = 1V$, $V_G = 0V$)	53.7 ms	1027.5 ms	
Gate tunable WSe_2	few layer	532 nm ($V_D = -1V$)	10.4 ms	9.8 ms	20
PEI doping WSe_2	90 nm	520 nm ($V_D = 0V$, $V_G = 40V$)	200 μs	16 μs	27
Ar and O_2 plasma doping and optimized metal work functions in WSe_2	20 nm	450 nm	264 ns	552 ns	28
Laser-induced p-type WSe_2	20 nm	633 nm ($V_D = 0V$, $V_G = 40V$)	136 μs	39μs	42
Oxidized WSe_2Pristine WSe_2	3.5 nm	685 nm ($V_D = 0V$, $V_G = 0V$)	306.72 μs	315.36 μs	This work

REFERENCES

[1] Yamamoto, M.; Dutta, S.; Aikawa, S.; Nakaharai, S.; Wakabayashi, K.; Fuhrer, M. S.; Ueno, K.; Tsukagoshi, K. Self-Limiting Layer-by-Layer Oxidation of Atomically Thin WSe_2. *Nano Letters* **2015**, *15*, 2067–2073, PMID: 25646637.

[2] Sivan, M.; Li, Y.; Veluri, H.; Zhao, Y.; Tang, B.; Wang, X.; Zamburg, E.; Leong, J. F.; Niu, J. X.; Chand, U.; Thean, A. V.-Y. All WSe_2 1T1R resistive RAM cell for future monolithic 3D embedded memory integration. *Nature Communications* **2019**, *10*, 5201.

[3] Lee, K.; Ngo, T. D.; Lee, S.; Shin, H.; Choi, M. S.; Hone, J.; Yoo, W. J. Effects of Oxygen Plasma Treatment on Fermi-Level Pinning and Tunneling at the Metal–Semiconductor Interface of WSe_2 FETs. *Advanced Electronic Materials* **2023**, *9*, 2200955.

[4] Chen, H.-Y.; Lin, J.-J.; Wong, S.-S.; Lin, Z.-Y.; Hsieh, Y.-C.; Chang, K.-E.; Wu, C.-L.; Watanabe, K.; Taniguchi, T.; Chen, T.-M.; Smith, L. W. Locally Doped Transferred Contacts for WSe_2 Transistors. *ACS Applied Electronic Materials* **2024**, *6*, 8319–8327.

[5] Wang, S.; Zhao, W.; Giustiniano, F.; Eda, G. Effect of oxygen and ozone on p-type doping of ultra-thin WSe_2 and $MoSe_2$ field effect transistors. *Phys. Chem. Chem. Phys.* **2016**, *18*, 4304–4309.

[6] Kato, R.; Uchiyama, H.; Nishimura, T.; Ueno, K.; Taniguchi, T.; Watanabe, K.; Chen, E.; Nagashio, K. p-Type Conversion of WS_2 and WSe_2 by Position-Selective Oxidation Doping and Its Application in Top Gate Transistors. *ACS Applied Materials & Interfaces* **2023**, *15*, 26977–26984, PMID: 37222246.

[7] Yamamoto, M.; Nakaharai, S.; Ueno, K.; Tsukagoshi, K. Self-Limiting Oxides on WSe_2 as Controlled Surface Acceptors and Low-Resistance Hole Contacts. *Nano Letters* **2016**, *16*, 2720–2727, PMID: 26963588.

[8] Borah, A.; Nipane, A.; Choi, M. S.; Hone, J.; Teherani, J. T. Low-Resistance p-Type Ohmic Contacts to Ultrathin WSe_2 by Using a Monolayer Dopant. *ACS Applied Electronic Materials* **2021**, *3*, 2941–2947.

[9] Nipane, A. et al. Damage-Free Atomic Layer Etch of WSe_2: A Platform for Fabricating Clean Two-Dimensional Devices. *ACS Applied Materials & Interfaces* **2021**, *13*, 1930–1942, PMID: 33351577.

[10] Li, Z.; Yang, S.; Dhall, R.; Kosmowska, E.; Shi, H.; Chatzakis, I.; Cronin, S. B. Layer Control of WSe_2 via Selective Surface Layer Oxidation. *ACS Nano* **2016**, *10*, 6836–6842, PMID: 27391161.

[11] Park, J. H.; Vishwanath, S.; Liu, X.; Zhou, H.; Eichfeld, S. M.; Fullerton-Shirey, S. K.; Robinson, J. A.; Feenstra, R. M.; Furdyna, J.; Jena, D.; Xing, H. G.; Kummel, A. C. Scanning Tunneling Microscopy and Spectroscopy of Air Exposure Effects on Molecular Beam Epitaxy Grown WSe_2 Monolayers and Bilayers. *ACS Nano* **2016**, *10*, 4258–4267, PMID: 26991824.

[12] Abualnaja, F.; Chu, G.; Lin, X.; Chen, H.-Y.; Lin, J.-J.; Joyce, H. J.; Hofmann, S.; Smith, L. W.; Alexander-Webber, J. A. Spectroscopic imaging ellipsometry for spatially resolved mapping of layer-by-layer oxidation in WSe_2. *Applied Physics Letters* **2026**, *128*, 211601.

[13] Das, S.; Chen, H.-Y.; Penumatcha, A. V.; Appenzeller, J. High Performance Multilayer MoS_2 Transistors with Scandium Contacts. *Nano Letters.* **2013**, *13*, 100–105.

[14] Mitta, S. B.; Ali, F.; Yang, Z.; Moon, I.; Ahmed, F.; Yoo, T. J.; Lee, B. H.; Yoo, W. J. Gate-Modulated Ultrasensitive Visible and Near-Infrared Photodetection of Oxygen Plasma-Treated WSe_2 Lateral pn-Homojunctions. *ACS Applied Materials & Interfaces* **2020**, *12*, 23261–23271, PMID: 32347702.

[15] Cui, X. et al. Low-Temperature Ohmic Contact to Monolayer MoS_2 by van der Waals Bonded Co/h-BN Electrodes. *Nano Letters* **2017**, 4781–4786.

[16] Allain, A.; Kang, J.; Banerjee, K.; Kis, A. Electrical contacts to two-dimensional semiconductors. *Nature Materials.* **2015**, *14*.

[17] Mitta, S. B.; Choi, M. S.; Nipane, A.; Ali, F.; Kim, C.; Teherani, J. T.; Hone, J.; Yoo, W. J. Electrical characterization of 2D materials-based field-effect transistors. *2D Materials* **2020**, *8*, 012002.

[18] Abbasi, I. H.; Albrow-Owen, T.; Abualnaja, F.; Mouthaan, R.; Potočnik, T.; Christopher, P. J.; Tan, H. H.; Jagadish, C.; Wong-Leung, J.; Alexander-Webber, J. A.; Joyce, H. J. InAs nanowire transistor pairs as NMOS inverters. *Nanotechnology* **2025**, *36*, 245202.

[19] Viscardi, L.; Faella, E.; Intonti, K.; Giubileo, F.; Demontis, V.; Prete, D.; Zannier, V.; Sorba, L.; Rossella, F.; Di Bartolomeo, A. Temperature behavior and logic circuit applications of InAs nanowire-based field-effect transistors. *Materials Science in Semiconductor Processing* **2024**, *173*, 108167.

[20] Groenendijk, D. J.; Buscema, M.; Steele, G. A.; Michaelis de Vasconcellos, S.; Bratschitsch, R.; van der Zant, H. S. J.; Castellanos-Gomez, A. Photovoltaic and Photothermoelectric Effect in a Double-Gated WSe_2 Device. *Nano Letters* **2014**, *14*, 5846–5852, PMID: 25232893.

[21] Niu, Y.; Gonzalez-Abad, S.; Frisenda, R.; Marauhn, P.; Drüppel, M.; Gant, P.; Schmidt, R.; Taghavi, N. S.; Barcons, D.; Molina-Mendoza, A. J.; De Vasconcellos, S. M.; Bratschitsch, R.; Perez De Lara, D.; Rohlfing, M.; Castellanos-Gomez, A. Thickness-Dependent Differential Reflectance Spectra of Monolayer and Few-Layer MoS_2, $MoSe_2$, WS_2 and WSe_2. *Nanomaterials* **2018**, *8*.

[22] Zhao, Q.; Wang, W.; Carrascoso-Plana, F.; Jie, W.; Wang, T.; Castellanos-Gomez, A.; Frisenda, R. The role of traps in the photocurrent generation mechanism in thin InSe photodetectors. *Mater. Horiz.* **2020**, *7*, 252–262.

[23] Liu, M.; Wei, J.; Qi, L.; An, J.; Liu, X.; Li, Y.; Shi, Z.; Li, D.; Novoselov, K. S.; Qiu, C.-W.; Li, S. Photogating-assisted tunneling boosts the responsivity and speed of heterogeneous WSe2/Ta2NiSe5 photodetectors. *Nature Communications* **2024**, *15*.

[24] Polyanskiy, M. N. Refractiveindex.info database of optical constants. *Scientific Data* **2024**, *11*, 94.

[25] Hsu, C.; Frisenda, R.; Schmidt, R.; Arora, A.; de Vasconcellos, S. M.; Bratschitsch, R.; van der Zant, H. S. J.; Castellanos-Gomez, A. Thickness-Dependent Refractive Index of 1L, 2L, and 3L MoS_2, $MoSe_2$, WS_2, and WSe_2. *Advanced Optical Materials* **2019**, *7*, 1900239.

[26] Gu, H.; Song, B.; Fang, M.; Hong, Y.; Chen, X.; Jiang, H.; Ren, W.; Liu, S. Layer-dependent dielectric and optical properties of centimeter-scale 2D WSe_2: evolution from a single layer to few layers. *Nanoscale* **2019**, *11*, 22762–22771.

[27] Tang, Y.; Wang, Z.; Wang, P.; Wu, F.; Wang, Y.; Chen, Y.; Wang, H.; Peng, M.; Shan, C.; Zhu, Z.; Qin, S.; Hu, W. WSe_2 Photovoltaic Device Based on Intramolecular p–n Junction. *Small* **2019**, *15*, 1805545.

[28] Zhang, Y.; Ma, K.; Zhao, C.; Hong, W.; Nie, C.; Qiu, Z.-J.; Wang, S. An Ultrafast WSe2 Photodiode Based on a Lateral p-i-n Homojunction. *ACS Nano* **2021**, *15*, 4405–4415, PMID: 33587610.

[29] Kim, Y.; Kim, T.; Jeong, W.; Jeong, M. S.; Kim, E. K. A high-performance logic inverter achieved using mixed-dimensional WSe_2/n^+-Si and MoS_2/p^+-Si junction field-effect transistors. *J. Mater. Chem. C* **2023**, *11*, 15649–15656.

[30] Du, W.; Jia, X.; Cheng, Z.; Xu, W.; Li, Y.; Dai, L. Low-power-consumption CMOS inverter array based on CVD-grown p-$MoTe_2$ and n-MoS_2. *iScience* **2021**, *24*, 103491.

[31] Wang, J.; Guo, X.; Yu, Z.; Ma, Z.; Liu, Y.; Lin, Z.; Chan, M.; Zhu, Y.; Wang, X.; Chai, Y. Low-Power Complementary Inverter with Negative Capacitance 2D Semiconductor Transistors. *Advanced Functional Materials* **2020**, *30*, 2003859.

[32] Ali, A.; Schrade, M.; Xing, W.; Vullum, P. E.; Koybasi, O.; Taniguchi, T.; Watanabe, K.; Belle, B. D. Two-Dimensional Heterostructure Complementary Logic Enabled by Optical Writing. *Small Science* **2024**, *4*, 2300319.

[33] Zhang, H.; Li, C.; Wang, J.; Hu, W.; Zhang, D. W.; Zhou, P. Complementary Logic with Voltage Zero-Loss and Nano-Watt Power via Configurable MoS_2/WSe_2 Gate. *Advanced Functional Materials* **2018**, *28*, 1805171.

[34] Tosun, M.; Chuang, S.; Fang, H.; Sachid, A. B.; Hettick, M.; Lin, Y.; Zeng, Y.; Javey, A. High-Gain Inverters Based on WSe_2 Complementary Field-Effect Transistors. *ACS Nano* **2014**, *8*, 4948–4953, PMID: 24684575.

[35] Wang, A.; Huang, H.; Sun, S.; He, Y.; Yang, Z.; Pan, J.; Li, Z.; Pan, D.; Zou, B.; Liao, L. Controlling the Polarity of WSe_2 FETs by Interface Engineering for High-Gain CMOS. *ACS Applied Nano Materials* **2024**, *7*, 5507–5512.

[36] Kong, L.; Zhang, X.; Tao, Q.; Zhang, M.; Dang, W.; Li, Z.; Feng, L.; Liao, L.; Duan, X.; Liu, Y. Doping-free complementary WSe_2 circuit via van der Waals metal integration. *Nature Communications* **2020**, *11*, 1866.

[37] Pendurthi, R. et al. Monolithic three-dimensional integration of complementary two-dimensional field-effect transistors. *Nature Nanotechnology* **2024**, *19*, 970–977.

[38] Ngo, T. D.; Yang, Z.; Lee, M.; Ali, F.; Moon, I.; Kim, D. G.; Taniguchi, T.; Watanabe, K.; Lee, K.-Y.; Yoo, W. J. Fermi-Level Pinning Free High-Performance 2D CMOS Inverter Fabricated with Van Der Waals Bottom Contacts. *Advanced Electronic Materials* **2021**, *7*, 2001212.

[39] Shin, H.; Taqi, M.; Ali, F.; Lee, S.; Choi, M. S.; Kim, C.; Lee, B.-H.; Liu, X.; Sun, J.; Oh, B.; Yoo, W. J. Self-Powered 2D $MoS_2/WO_x/WSe_2$ Heterojunction Photodetector Realized by Oxygen Plasma Treatment. *Advanced Materials Interfaces* **2022**, *9*, 2201785.

[40] Yue, D.; Ju, X.; Hu, T.; Rong, X.; Liu, X.; Liu, X.; Ng, H. K.; Chi, D.; Wang, X.; Wu, J. Homogeneous in-plane WSe_2 P-N junctions for advanced optoelectronic devices. *Nanoscale* **2023**, *15*, 4940–4950.

[41] Li, M.-Z.; Hung, T. Y. T.; Yun, W.-S.; Sathaiya, D. M.; Chou, S.-A.; Liew, S. L.; Yang, Y.-M.; Lin, K.-I.; Lee, T.-Y.; Cheng, C.-C.; Wu, C.-C.; Radu, I. P.; Lin, M.-T. Top-Gated P-MOSFET with CVD-Grown WSe_2 Channels via Self-Aligned WOx Conversion for Spacer Doping. *Nano Letters* **2025**, *25*, 7037–7043, PMID: 40232827.

[42] Chen, J.; Wang, Q.; Sheng, Y.; Cao, G.; Yang, P.; Shan, Y.; Liao, F.; Muhammad, Z.; Bao, W.; Hu, L.; Liu, R.; Cong, C.; Qiu, Z.-J. High-Performance WSe2 Photodetector Based on a Laser-Induced p–n Junction. *ACS Applied Materials & Interfaces* **2019**, *11*, 43330–43336, PMID: 31659890.
